# Case Report: Differentiating hepatic desmoplastic small round cell tumor from hydatidosis in a school-aged boy: the role of contrast-enhanced and interventional ultrasound

**DOI:** 10.3389/fonc.2025.1493237

**Published:** 2025-03-13

**Authors:** Zehui Gou, Hualin Yan, Boyang Yu, Xiaolong Xie, Bo Xiang, Juxian Liu, Yan Luo

**Affiliations:** ^1^ Department of Medical Ultrasound, West China Hospital, Sichuan University, Chengdu, China; ^2^ Department of Pediatric Surgery, West China Hospital, Sichuan University, Chengdu, China

**Keywords:** desmoplastic small round cell tumor, contrast-enhanced ultrasonography (CEUS), interventional ultrasound, hydatidosis, pediatrics

## Abstract

**Background:**

Desmoplastic small round cell tumor (DSRCT) is a rare and highly aggressive malignant neoplasm, typically associated with poor prognosis. It predominantly affects adolescents and young males, with a lower incidence in pediatric populations. Due to its rarity, our understanding of DSRCT remains limited, with only a small number of case reports available. The clinical presentation is often non-specific and varies depending on the extent of tumor invasion. Diagnosis relies primarily on histopathological evaluation through biopsy. Although imaging studies contribute to the diagnostic process, they often lack specificity. Nonetheless, certain imaging features can aid in refining differential diagnoses and assessing disease severity. Moreover, minimally invasive, image-guided tissue sampling plays a critical role in confirming the diagnosis through pathological analysis.

**Case presentation:**

A 7-year-old boy presented with abdominal distension and anorexia, without significant abdominal pain, fever, or jaundice. Physical examination revealed abdominal enlargement with hepatosplenomegaly. Laboratory tests showed abnormal liver function (AST 128 U/L, ALP 648 U/L, GGT 885 U/L) and an elevated CA-125 level (170 U/ml). An abdominal CT scan performed at a local hospital identified multiple round, low-density lesions in the liver, suggestive of echinococcosis. The patient was initially diagnosed with suspected echinococcosis and started on albendazole; however, his symptoms did not improve. Upon further evaluation at our institution, ultrasound imaging revealed multiple thick-walled, hyperechoic lesions in the liver with no significant blood flow signals. Contrast-enhanced ultrasound demonstrated that the solid components of the lesion exhibited significant enhancement during the early arterial phase, with rapid attenuation during the early portal venous phase. A metastatic malignant tumor was suspected, prompting a percutaneous biopsy under real-time enhanced ultrasound guidance. Histopathological examination revealed small round tumor cells infiltrating adjacent tissues. Fluorescence *in situ* hybridization (FISH) confirmed the diagnosis of DSRCT, based on the presence of an EWSR1-WT1 rearrangement. The patient subsequently underwent multimodal treatment, including chemotherapy and radiation therapy, and achieved disease-free survival at the six-month follow-up.

**Conclusions:**

Traditional ultrasound is a convenient, real-time, non-invasive, and radiation-free diagnostic tool, making it particularly well-suited for the diagnosis, screening, and clinical follow-up of focal liver lesions (FLLs) in pediatric patients. This modality enables real-time evaluation of the number, size, location, and morphology of FLLs while assisting in the differential diagnosis. Moreover, it facilitates the assessment of liver parenchyma involvement and portal vein structures. Color Doppler imaging provides valuable insights into the vascular characteristics of tumors, while contrast-enhanced ultrasound (CEUS) agents allow for real-time observation of dynamic tumor perfusion patterns, further refining differential diagnoses based on perfusion characteristics. Compared to the contrast agents used in CT or MRI—which may require sedation or carry risks of renal injury due to radiation exposure—ultrasound microbubble contrast agents are excreted via respiration and do not require sedation, making them especially suitable for pediatric patients. Additionally, ultrasound-guided biopsy is a well-established and reliable method for diagnosing liver lesions. However, the presence of extensive necrosis and the use of fine-needle biopsy can sometimes limit diagnostic accuracy. Incorporating CEUS before or during percutaneous biopsy can help optimize sampling site selection, thereby reducing the likelihood of false-negative results.

## 1 Introduction

Malignant focal liver lesions (FLLs) are common in adults but rare in pediatric populations (1–4). Due to their infrequency, knowledge regarding pediatric FLLs remains limited, creating challenges in their non-invasive detection and characterization, as well as in the selection of appropriate imaging modalities (3). In 2017, the European Federation of Societies for Ultrasound in Medicine and Biology (EFSUMB) recommended the use of contrast-enhanced ultrasound (CEUS) for the evaluation and follow-up of pediatric FLLs (5). The World Federation of Ultrasound in Medicine and Biology (WFUMB) similarly endorsed CEUS in 2020 as a child-friendly and effective diagnostic tool for FLLs, offering the advantage of avoiding unnecessary radiation exposure (6). However, to date, no pediatric-specific CEUS liver imaging reporting and data system (LI-RADS) standards have been established. The application of CT/MRI LI-RADS in children is limited by moderate sensitivity for hepatocellular carcinoma (HCC) and low specificity, though it demonstrates high sensitivity in detecting any type of malignant liver tumor, with a high negative predictive value (NPV) (7).

Based on clinical experience and a review of the literature, CEUS patterns in pediatric patients closely resemble those seen in adults, characterized by pronounced enhancement during the early arterial phase, followed by rapid attenuation in the early portal phase. These characteristics show good sensitivity and specificity for diagnosing malignant liver lesions (8, 9).

This article reports a rare case of malignant focal liver lesions (FLLs) in a pediatric patient. Initially misdiagnosed as cystic hydatid disease via CT imaging due to the lack of specific diagnostic criteria, the patient received albendazole treatment without symptomatic improvement. Subsequent multimodal diagnostic evaluation revealed key findings: Contrast-enhanced ultrasound (CEUS) suggested a metastatic malignant liver tumor, followed by a real-time CEUS-guided percutaneous biopsy that identified invasive small round tumor cells. Fluorescence *in situ* hybridization further identified an EWSR1-WT1 rearrangement, confirming a diagnosis of desmoplastic small round cell tumor (DSRCT). The patient was subsequently treated with a multimodal regimen of chemotherapy and radiation therapy, achieving disease-free survival at six months post-treatment.

## 2 Case description

**Figure 1:**
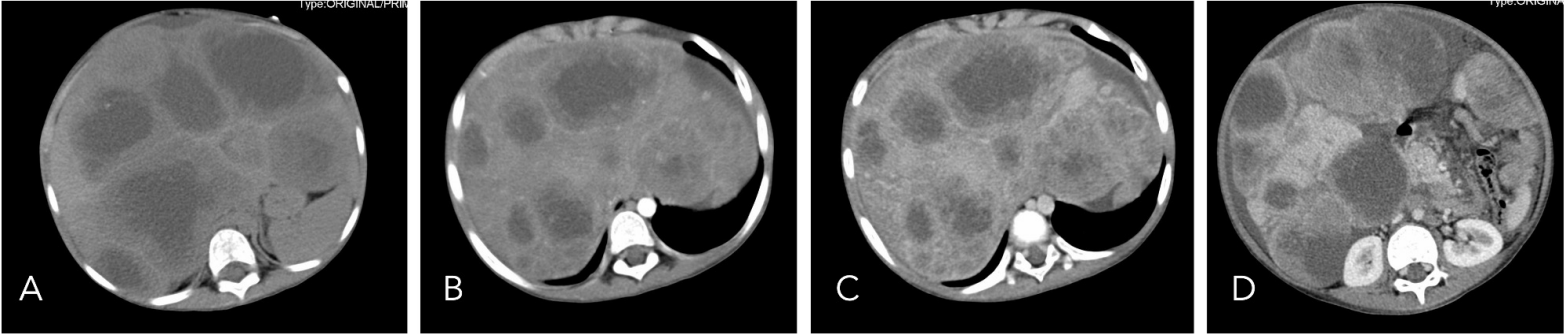
On non-contrast CT imaging **(A)**, the liver demonstrates abnormal morphology and significant enlargement in size. The hepatic parenchyma is diffusely infiltrated by multiple hypodense cystic nodules and masses, with the largest lesion measuring approximately 12.2 × 6.6 cm. On contrast-enhanced imaging [**(B)**: arterial phase, **(C)**: portal venous phase, **(D)**: delayed phase], these lesions exhibit peripheral rim enhancement with poorly defined margins. Focal areas of increased density are noted within the walls of some cysts, and certain lesions extend beyond the hepatic contour.

A 7-year-old male patient was admitted to a local hospital due to progressive abdominal distension and anorexia of six-month duration without any identifiable precipitating factors. There were no associated symptoms such as abdominal pain, fever, or jaundice. On physical examination, notable findings included abdominal distension and hepatosplenomegaly. Laboratory tests indicated abnormal liver function with elevated AST (128 U/L), ALP (648 U/L), and GGT (885 U/L), along with a raised CA-125 level (170 U/ml). An abdominal CT scan performed at a local hospital revealed multiple round low-density lesions in the liver, initially suggestive of echinococcosis (**Figure 1**). Based on this provisional diagnosis, the patient was started on albendazole treatment; however, after one month of treatment, there was no improvement in symptoms, which instead progressively worsened. Furthermore, the patient developed dyspnea over the preceding five days, leading to his urgent referral to our institution for further evaluation and management.

**Figure 2:**
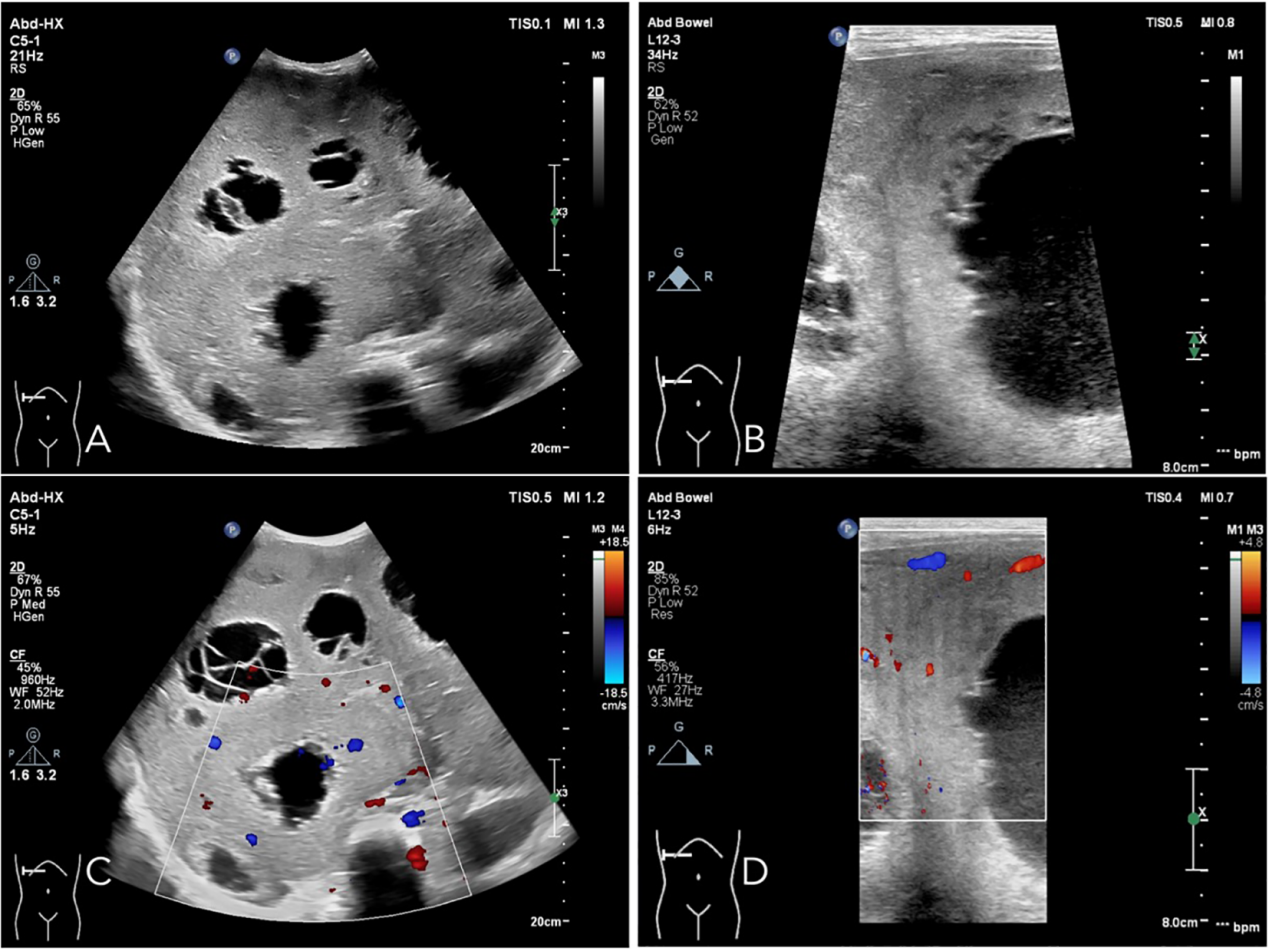
Ultrasound examination reveals that the liver demonstrates abnormal morphology and is significantly enlarged. The echogenicity of the hepatic parenchyma is heterogeneous, characterized by multiple thick-walled cystic-solid mixed lesions of varying sizes, which exhibit indistinct boundaries and irregular shapes. Some lesions show segmentation features. The largest lesion, located in the right lobe of the liver, measures approximately 10.0 × 7.0 × 8.0 cm, with a cystic wall thickness ranging from 1 to 2 cm **(A, B)**. Vascular flow signals are detectable within the solid components of the cystic wall **(C, D)**.

**Figure 3:**
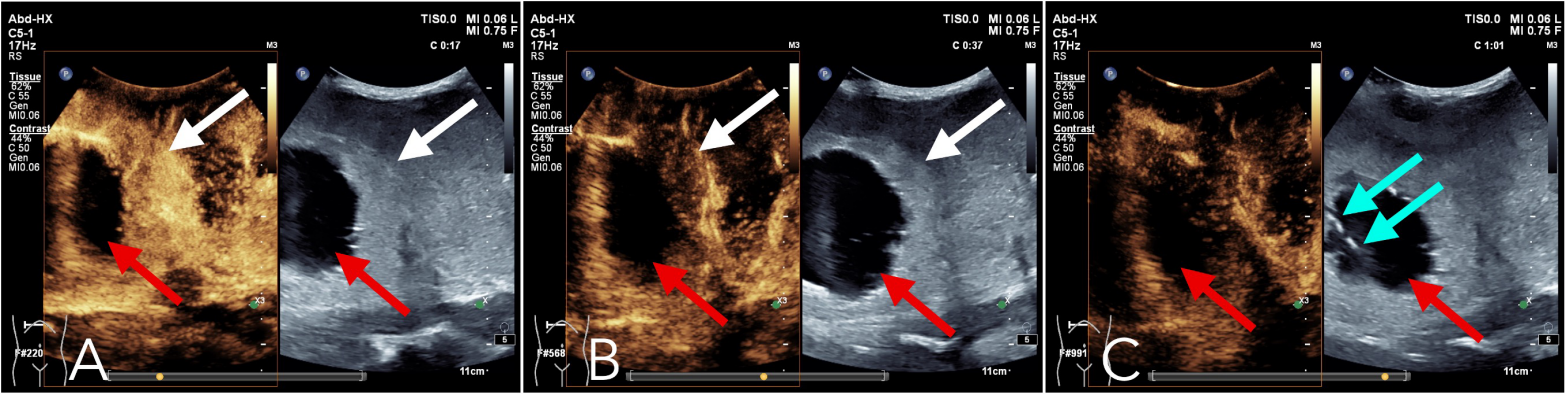
Contrast-enhanced ultrasound: Following intravenous administration of the contrast agent Sonovue, the liver lesions exhibited a pronounced enhancement in the early arterial phase **(A)**, primarily within the solid components surrounding the lesion (as indicated by the white arrows). In the subsequent early portal venous phase **(B)**, this enhancement rapidly diminished (as indicated by the white arrows). By the parenchymal phase **(C)**, the enhancement effect had further decreased. Throughout the arterial, portal venous, and parenchymal phases **(A-C)**, no significant enhancement was observed in the fluid-filled central region of the lesion (as indicated by the red arrows). The blue arrow in **(C)** denotes the position of the needle tip during the parenchymal phase biopsy.

Upon referral to our institution, further evaluation using ultrasound imaging revealed abnormal liver morphology, with significant enlargement extending into the right iliac fossa. The hepatic parenchyma exhibited heterogeneous echogenicity, and numerous thick-walled, mixed cystic-solid lesions of varying sizes were identified within the liver, characterized by ill-defined borders and irregular configurations. Some lesions displayed a septate architecture. The largest lesion, located in the right lobe of the liver, measured approximately 10.0 x 7.0 x 8.0 cm, with cystic wall thickness ranging from 1 to 2 cm. Blood flow signals were detected within the solid components of the cystic wall (**Figure 2**). Following intravenous administration of SonoVue, the solid components exhibited significant enhancement during the early arterial phase, with rapid attenuation during the early portal venous phase. No enhancement was observed in the central fluid-filled regions of the lesions (**Figure 3**).

Given the findings, a metastatic malignant tumor was suspected, and a percutaneous biopsy was performed under enhanced ultrasound guidance. Using a coaxial needle biopsy technique, a 17G coaxial needle was advanced through the normal liver parenchyma to the periphery of the lesion. The needle core was then retracted, and an 18G hollow needle was attached to the biopsy gun ensuring precise targeting, the needle tip was positioned at the forefront of the lesion in areas exhibiting significant enhancement on ultrasound contrast imaging (**Figure 3C**). Three tissue specimens were successfully obtained from the solid component of the lesion. Following the biopsy, the needle tract was occluded using a gelatin sponge slurry. The gelatin sponge particles were loaded into an injection syringe, while another syringe containing 2 mL of normal saline was connected to the first via a three-way valve. The Tessari technique was employed to transform the gelatin sponge into a viscous paste. Subsequently, the temporary occlusion agent was administered into the needle tract through a coaxial needle and gradually retracted until it reached the liver surface.

**Figure 4:**
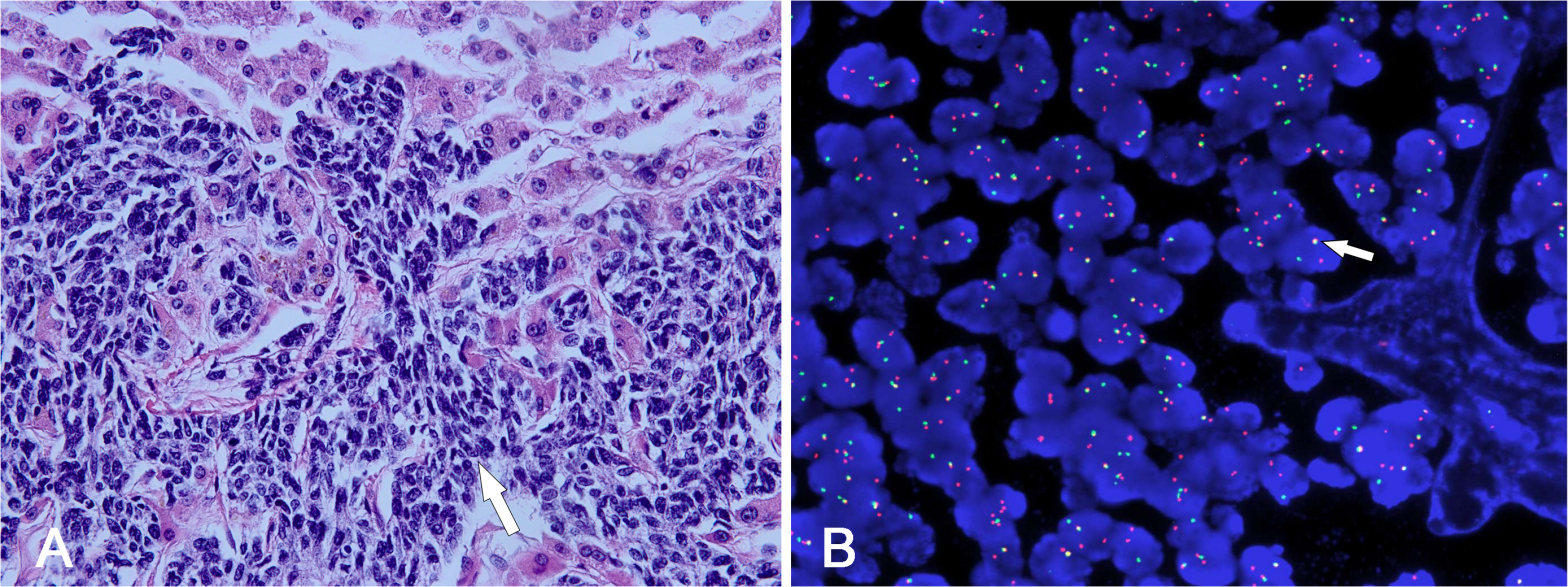
**(A)** The tumor cells were typically uniform small neoplastic round. They were characterized by various-sized nests invading into hepatic parenchyma (white arrow). **(B)** FISH study demonstrated separate red and green signals, indicating EWSR1-WT1 gene rearrangement (white arrow).

Histopathological analysis revealed the presence of small round tumor cells with invasive characteristics involving adjacent tissues (**Figure 4A**). Fluorescence *in situ* hybridization (FISH) confirmed a diagnosis of desmoplastic small round cell tumor (DSRCT) through the detection of EWSR1-WT1 rearrangement (**Figure 4B**). The patient subsequently underwent a comprehensive multimodal treatment regimen comprising chemotherapy and radiotherapy. Following 43 days of intensive treatment, the patient reported a marked alleviation of symptoms and was subsequently discharged from the hospital. During the 6-month follow-up period, the patient remained asymptomatic and survived without significant complications.

## 3 Discussion

DSRCT is an extremely rare aggressive sarcoma, with a global incidence rate lower than one in a million. It is predominantly manifested in adolescents and young adults (with a median age of 20 years), and cases primarily emerging in the liver are exceedingly rare, mostly documented as individual cases in the literature (1, 3, 10). In China, reports are scarce, and only sporadic case reports are available at present, lacking definite epidemiological statistics. Early diagnosis depends on imaging and pathological examinations. However, the imaging features on contrast-enhanced ultrasound (CEUS) or CT imaging are not specific, so getting a biopsy through intervention methods is important for precise early diagnosis. The prognosis of DSCRT is unfavorable.

While liver metastases commonly present as low-density lesions on CT imaging, the appearance of multiple ring-shaped high-density shadows, as seen in DSRCT, is exceptionally rare and can be easily misdiagnosed as hydatid disease. The ultrasound characteristics of DSRCT are not well-documented, but the echogenicity of the lesions may vary depending on the degree of necrosis and hemorrhage, with some cases also showing microcalcifications. Doppler imaging typically reveals an uneven blood supply to the tumors (11).

According to the European Society of Ultrasound in Medicine (EFSUMB) position statement, contrast-enhanced ultrasound (CEUS) is particularly useful for characterizing liver lesions in pediatric patients, one of the most common CEUS applications in this demographic (6). Although CEUS LI-RADS does not specifically address pediatric cases, some reports suggest that low contrast enhancement during the portal vein phase, following arterial phase imaging, may serve as a highly specific marker for malignancy in children (1, 4, 12, 13). Moreover, the safety of ultrasound contrast agents (UCAs) in pediatric populations has been confirmed to be comparable to that in adults, minimizing the risks of radiation exposure (5, 14).

To our knowledge, this is the first documented case detailing the CEUS findings in a patient with liver DSRCT. The CEUS exhibited a characteristic “wash-in/wash-out” pattern that effectively distinguished the malignant hepatic tumor from hydatid disease (15). Additionally, ultrasound-guided percutaneous liver biopsy proved to be a safe and efficient technique for obtaining diagnostic tissue samples, with a low complication rate (16–18). However, in the presence of large necrotic areas and fine-needle biopsy, diagnostic accuracy may be compromised. Utilizing CEUS to identify optimal sampling sites prior to or during the biopsy procedure can help mitigate the risk of false-negative results, thereby improving diagnostic accuracy for DSRCT. In this case, we used a coaxial needle biopsy technique to obtain tissue samples. The liver capsule was only penetrated once during the procedure, and the needle was guided to the target lesion area under real-time ultrasound monitoring to avoid any important blood vessels or critical structures. After the biopsy, we used gelatin sponge to seal the puncture site, minimizing the risk of postoperative bleeding. Based on a thorough review of the literature and our extensive clinical experience, we assert that the technique of needle canal occlusion represents a safe and viable intervention applicable in the vast majority of clinical contexts, exhibiting a high degree of reliability (18–21).

In this case, the initial misinterpretation of CT images led to a misdiagnosis of DSRCT as echinococcosis, resulting in ineffective treatment and disease progression for the patient. To prevent such misdiagnoses in the future, we conducted a thorough review of the literature on DSRCT and echinococcosis. Based on our diagnostic and therapeutic experience with this case, we summarized the key imaging features of both conditions to aid in differential diagnosis in **Table 1** (22–24).

**Table 1:** Key imaging features of dsrct and hydatid disease.

Feature	DSRCT	Hydatid Disease
Location	Abdomen/pelvis (peritoneal spread)	Liver (70%), lungs, peritoneal cavity
Morphology	Lobulated soft tissue mass, multiple nodules	Cystic (CE type) or infiltrative (alveolar type)
Contrast-enhanced CT	Heterogeneous enhancement, central necrosis	Cyst wall enhancement, no internal enhancement
Calcification	~20%, punctate	Cystic: 20-30%; Alveolar: 50% (extensive)
CEUS Performance	Hyperenhancement in solid areas, non-enhancing necrosis	Hyperenhancing cyst wall, no internal enhancement
Diffusion restriction (DWI-MRI)	Marked (low ADC values)	Absent (free diffusion of cystic fluid)
Laboratory Markers	Non-specific; requires immunohistochemistry (WT1+)	Serum IgG antibody positive (ELISA/Western blot)
Clinical context	Adolescents/young adults, abdominal pain/mass	History of livestock exposure, asymptomatic or compressive symptoms

This case highlights the critical role of integrating CEUS and ultrasound-guided percutaneous liver biopsy in rectifying an initial misdiagnosis of liver hydatid cyst disease and ultimately confirming a diagnosis of DSRCT. These two advanced imaging modalities proved invaluable, illustrating their significant clinical utility in diagnosing rare and complex liver conditions in pediatric patients.

## 4 Conclusions

Traditional ultrasound remains a highly advantageous diagnostic tool for pediatric focal liver lesions (FLLs) due to its convenience, real-time imaging capability, non-invasiveness, and absence of radiation. This technique allows for the comprehensive assessment of FLLs in terms of number, size, location, and morphology, and aids in refining differential diagnoses. It also facilitates the evaluation of liver parenchyma involvement and portal vein structures. Color Doppler imaging enhances our understanding of tumor vascularity, while contrast-enhanced ultrasound (CEUS) offers dynamic insights into tumor perfusion patterns, contributing to more accurate differential diagnoses.

Unlike contrast agents used in CT or MRI—which may entail risks such as sedation or renal injury from radiation exposure—ultrasound microbubble contrast agents are excreted via respiration and do not require sedation, making them especially suitable for pediatric populations. Additionally, ultrasound-guided biopsy has proven to be a reliable method for diagnosing liver lesions; however, the presence of extensive necrotic areas and the use of fine-needle biopsies may reduce diagnostic accuracy. CEUS, when utilized prior to or during percutaneous biopsy, can enhance the selection of optimal sampling points, thereby minimizing the risk of false-negative results and improving diagnostic outcomes.

## Data Availability

The original contributions presented in the study are included in the article/supplementary material. Further inquiries can be directed to the corresponding authors.

## References

[1] Dong Y Cekuolis A Schreiber-Dietrich D Augustiniene R Schwarz S Möller K. Review on pediatric Malignant focal liver lesions with imaging evaluation: part I. Diagnostics (Basel). (2023) 13(23):3568. doi: 10.3390/diagnostics13233568 38066809 PMC10706220

[2] Hatanaka KC Takakuwa E Hatanaka Y Suzuki A IIzuka S Tsushima N. Desmoplastic small round cell tumor of the parotid gland-report of a rare case and a review of the literature. Diagn Pathol. (2019) 14:43. doi: 10.1186/s13000-019-0825-1 31103034 PMC6525968

[3] Morani AC Bathala TK Surabhi VR Yedururi S Jensen CT Huh WW. Desmoplastic small round cell tumor: imaging pattern of disease at presentation. AJR Am J roentgenology. (2019) 212:W45–w54. doi: 10.2214/AJR.18.20179 30673334

[4] Dong Y Cekuolis A Schreiber-Dietrich D Augustiniene R Schwarz S Möller K. Review on pediatric Malignant focal liver lesions with imaging evaluation: part II. Diagnostics (Basel). (2023) 13(24):3659. doi: 10.3390/diagnostics13243659 38132242 PMC10743166

[5] Sidhu PS Cantisani V Deganello A Dietrich CF Duran C Franke D. Role of contrast-enhanced ultrasound (CEUS) in paediatric practice: an EFSUMB position statement. Ultraschall Med. (2017) 38:33–43. doi: 10.1055/s-0042-110394 27414980

[6] Dietrich CF Nolsøe CP Barr RG Berzigotti A Burns PN Cantisani V. Guidelines and good clinical practice recommendations for contrast-enhanced ultrasound (CEUS) in the liver-update 2020 WFUMB in cooperation with EFSUMB, AFSUMB, AIUM, and FLAUS. Ultrasound Med Biol. (2020) 46:2579–604. doi: 10.1016/j.ultrasmedbio.2020.04.030 32713788

[7] Ludwig DR Romberg EK Fraum TJ Rohe E Fowler KJ Khanna G. Diagnostic performance of Liver Imaging Reporting and Data System (LI-RADS) v2017 in predicting Malignant liver lesions in pediatric patients: a preliminary study. Pediatr Radiol. (2019) 49:746–58. doi: 10.1007/s00247-019-04358-9 31069473

[8] Anupindi SA Biko DM Ntoulia A Poznick L Morgan TA Darge K. Contrast-enhanced US assessment of focal liver lesions in children. Radiographics. (2017) 37:1632–47. doi: 10.1148/rg.2017170073 29019750

[9] Dietrich CF Nolsøe CP Barr RG Berzigotti A Burns PN Cantisani V. Guidelines and good clinical practice recommendations for contrast enhanced ultrasound (CEUS) in the liver - update 2020 - WFUMB in cooperation with EFSUMB, AFSUMB, AIUM, and FLAUS. Ultraschall Med. (2020) 41:562–85. doi: 10.1055/a-1177-0530 32707595

[10] Gerald WL Haber DA. The EWS-WT1 gene fusion in desmoplastic small round cell tumor. Semin Cancer Biol. (2005) 15:197–205. doi: 10.1016/j.semcancer.2005.01.005 15826834

[11] Xu JS Luo Y Liu JX Li DY. Ultrasonic manifestations of desmoplastic small round cell tumor with multiple metastases: case report. Sichuan Da Xue Xue Bao Yi Xue Ban. (2020) 51:729–31. doi: 10.12182/20200960607 32975093

[12] Mostafa A Abramson Z Ghbrial M Biswas S Chan S Darji H. Contrast enhanced ultrasound of liver lesions in patients treated for childhood Malignancies. Cancer Imaging. (2024) 24:115. doi: 10.1186/s40644-024-00750-3 39210481 PMC11360734

[13] Han D Wang T Wang R Chen J Tang Y. Application of quantitative parameters of contrast-enhanced ultrasound in common benign and Malignant lesions in pediatric livers: A preliminary study. Diagnostics (Basel). (2023) 13(22):3443. doi: 10.3390/diagnostics13223443 37998580 PMC10670694

[14] Seitz K Strobel D. A milestone: approval of CEUS for diagnostic liver imaging in adults and children in the USA. Ultraschall Med. (2016) 37:229–32. doi: 10.1055/s-0042-107411 27276056

[15] Lantinga MA Gevers TJ Drenth JP. Evaluation of hepatic cystic lesions. World J Gastroenterol. (2013) 19:3543–54. doi: 10.3748/wjg.v19.i23.3543 PMC3691048 23801855

[16] Alturkistani H Alsergani AH Alzeer M Alturkistani A Zaini R Bauones S. Ultrasound-guided percutaneous liver biopsy: A review of what operators need to know. Med (Baltimore). (2024) 103:e38673. doi: 10.1097/MD.0000000000038673 PMC11272270 39058859

[17] Dotan Y Carlebach M Zuckerman E Maruf M Schiff E. Delayed bleeding after percutaneous liver biopsy. Eur J Case Rep Intern Med. (2016) 3:000359. doi: 10.12890/2016_000359 30755857 PMC6346949

[18] Singhal S Dp M Inuganti S Botcha S Deepashree DT Uthappa MC. Percutaneous ultrasound-guided plugged liver biopsy - a single-centre experience. Pol J Radiol. (2021) 86:e239–e45. doi: 10.5114/pjr.2021.105852 PMC8147717 34093921

[19] Tobin MV Gilmore IT. Plugged liver biopsy in patients with impaired coagulation. Dig Dis Sci. (1989) 34:13–5. doi: 10.1007/BF01536147 2910671

[20] Atar E Ben Ari Z Bachar GN Amlinski Y Neyman C Knizhnik M. A comparison of transjugular and plugged-percutaneous liver biopsy in patients with contraindications to ordinary percutaneous liver biopsy and an “in-house” protocol for selecting the procedure of choice. Cardiovasc Intervent Radiol. (2010) 33:560–4. doi: 10.1007/s00270-009-9743-z 19908088

[21] Kamphuisen PW Wiersma TG Mulder CJ de Vries RA. Plugged-percutaneous liver biopsy in patients with impaired coagulation and ascites. Pathophysiol Haemost Thromb. (2002) 32:190–3. doi: 10.1159/000070426 12759521

[22] Brunetti E Kern P Vuitton DA Writing Panel for the W-I. Expert consensus for the diagnosis and treatment of cystic and alveolar echinococcosis in humans. Acta Trop. (2010) 114:1–16. doi: 10.1016/j.actatropica.2009.11.001 19931502

[23] Iyer RS Schaunaman G Pruthi S Finn LS. Imaging of pediatric desmoplastic small-round-cell tumor with pathologic correlation. Curr Probl Diagn Radiol. (2013) 42:26–32. doi: 10.1067/j.cpradiol.2012.05.004 23146167

[24] Kantarci M Bayraktutan U Karabulut N Aydinli B Ogul H Yuce I. Alveolar echinococcosis: spectrum of findings at cross-sectional imaging. Radiographics. (2012) 32:2053–70. doi: 10.1148/rg.327125708 23150858

